# Relationship between hospital volume and short-term outcomes: a nationwide population-based study including 75,280 rectal cancer surgical procedures

**DOI:** 10.18632/oncotarget.24699

**Published:** 2018-03-30

**Authors:** Salvatore Pucciarelli, Manuel Zorzi, Nicola Gennaro, Francesco Marchegiani, Andrea Barina, Massimo Rugge, Matteo Zuin, Alessandro Perin, Isacco Maretto, Francesca Bergamo, Caterina Boso, Emanuele Damiano Luca Urso, Patrick Frambach, Maria Chiara Corti

**Affiliations:** ^1^ Department of Surgical, Oncological and Gastroenterological Sciences, University of Padua, Padua, Italy; ^2^ Regional Health Service, Veneto Tumor Registry, Veneto Region, Padua, Italy; ^3^ Regional Health Service, Epidemiology Unit, Veneto Region, Padua, Italy; ^4^ Department of Medicine DIMED, Pathology and Cytopathology Unit, University of Padua, Padua, Italy; ^5^ Medical Oncology 1, Veneto Institute of Oncology IOV – IRCCS, Padua, Italy; ^6^ Radiation Oncology, Veneto Institute of Oncology IOV – IRCCS, Padua, Italy

**Keywords:** rectal cancer, hospital volume, volume-outcome relationship, short-term outcomes, population study

## Abstract

There is growing interest on the potential relationship between hospital volume (HV) and outcomes as it might justify the centralization of care for rectal cancer surgery.

From the National Italian Hospital Discharge Dataset, data on 75,280 rectal cancer patients who underwent elective major surgery between 2002 and 2014 were retrieved and analyzed. HV was grouped into tertiles: low-volume performed 1-12, while high-volume hospitals performed 33+ procedures/year. The impact of HV on in-hospital mortality, abdominoperineal resection (APR), 30-day readmission, and length of stay (LOS) was assessed. Risk factors were calculated using multivariate logistic regression.

The proportion of procedures performed in low-volume hospitals decreased by 6.7 percent (p<0.001). The rate of in-hospital mortality, APR and 30-day readmission was 1.3%, 16.3%, and 7.2%, respectively, and the median LOS was 13 days. The adjusted risk of in-hospital mortality (OR = 1.49, 95% CI = 1.25-1.78), APR (OR 1.10, 95%CI 1.02-1.19), 30-day readmission (OR 1.49, 95%CI 1.38-1.61), and prolonged LOS (OR 2.29, 95%CI 2.05-2.55) were greater for low-volume hospitals than for high-volume hospitals.

This study shows an independent impact of HV procedures on all short-term outcome measures, justifying a policy of centralization for rectal cancer surgery, a process which is underway.

## INTRODUCTION

Rectal cancer requires complex surgical procedures and a multidisciplinary approach particularly when the tumor is located in the mid-low rectum and is locally advanced. Such complexity makes the management of this tumor similar to other cancers for which a significant association between hospital volume (HV) and outcomes has been found and centralization has been suggested [1, 2]). Actually, previous positive experiences in some European countries [3–5] seem to support this process. However, such centralization is source of debate and robust evidence of the relationship between HV procedures and outcomes has not been clearly demonstrated yet. As randomized trials are not feasible on this topic, current evidence is mainly based on retrospective observational studies. Differences in data source, study design, patient selection, HV definition, and healthcare systems make the findings of published studies widely heterogeneous, even among systematic reviews and meta-analyses [6–9]. Moreover, some of the events (e.g., postoperative mortality) used to define an HV-outcome relationship occur rarely after rectal surgery. As a consequence, most studies are underpowered to capture statistically significant differences in postoperative mortality between low and high-volume hospitals. Further potential biases of previous studies derive from the use of either a single outcome or outcomes that are difficult to retrieve because they are underreported or omitted. Among the measures used to evaluate the relationship between HV and short-term outcomes, the postoperative mortality and morbidity, the rate of abdominoperineal resection (APR), the length of stay (LOS) and the rate of unplanned readmission are the most used in the medical literature.

Given the unfeasibility of prospective randomized trial, we hypothesize that studies taking into account multiple outcome measures may strongly contribute to the debate of centralization of rectal cancer surgery. The strengths of these studies should be the longitudinal observational design and the easily retrieved data from large population-based datasets.

In this setting, we aimed to investigate whether short-term outcome measures are associated with HV after elective major rectal cancer procedures.

## RESULTS

### Baseline patient characteristics by hospital volume

**Figure 1:**
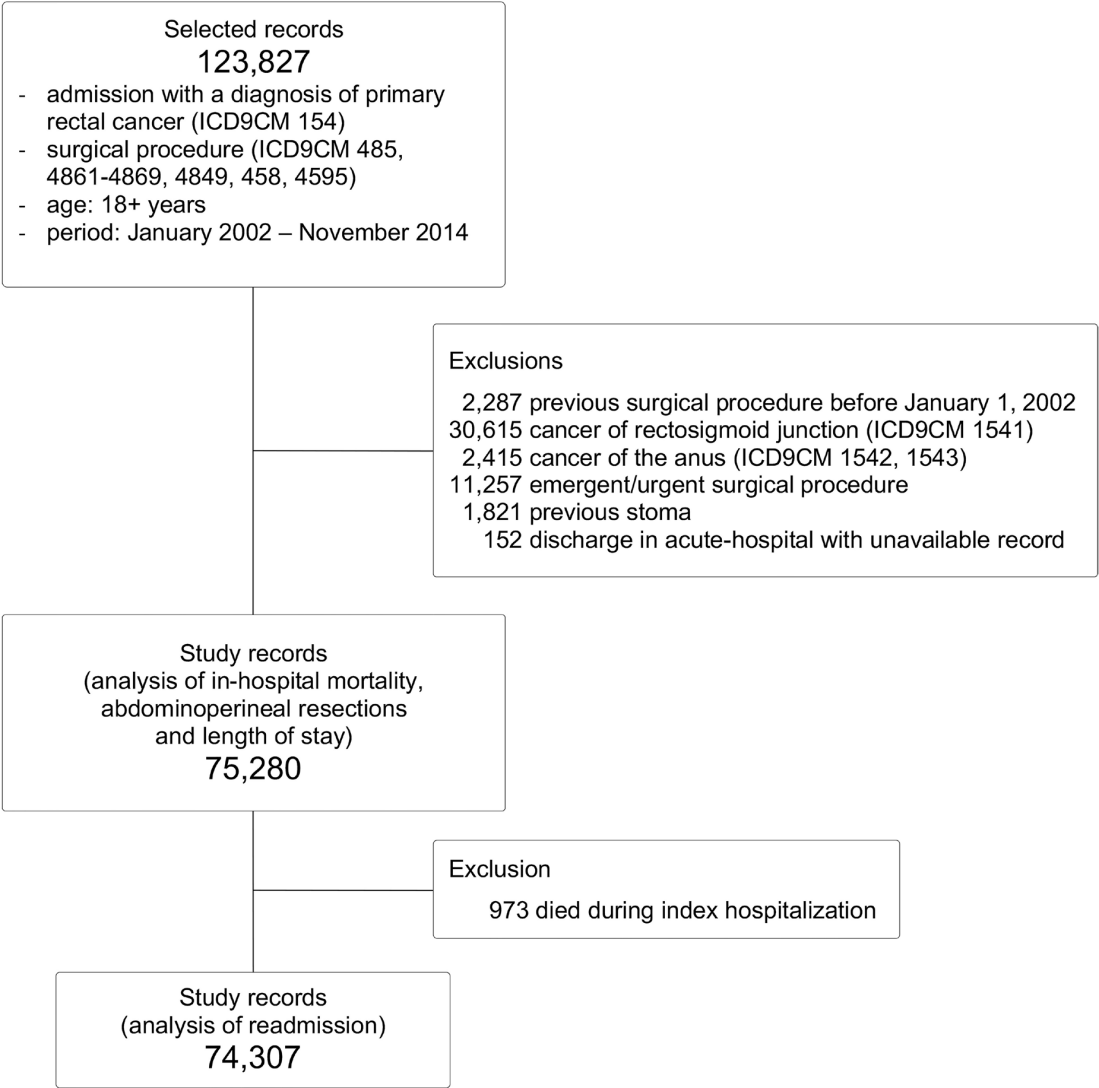
Flow chart of patients' selection

During the study period, 75,280 patients met the inclusion criteria. (Figure 1) Low-volume hospitals performed 1-12, medium-volume 13-31, and high-volume 32+ procedures per year. Compared with patients admitted to the high-volume, those admitted to the low-volume hospitals were more likely to be older (p<0.001) male (p=0.018), have a worse Charlson score (p<0.001), have received rectal surgery in the first period of the study (p<0.001), have a higher rate of stoma creation (p<0.001), and be treated with an open approach (p<0.001). Conversely, they were less likely to have been hospitalized in the year prior to the index surgery (p<0.001). (Table 1)

**Table 1:** Characteristics of patients and surgical approach by hospital volume

	All patients		Low volume (1 - 12)		Medium volume (13 - 31)		High volume (32+)		
	n	column %	n	column %	n	column %	n	column %	p-value
No. of Patients	75,280	100.0	25,576	100.0	24,213	100.0	25,491	100.0	
No. of hospitals/year	8,280	100.0	6,534	100.0	1,283	100.0	463	100.0	
**Age categories**									
18-49	4,863	6.5%	1,216	4.8%	1,428	5.9%	2,219	8.7%	p<0.001
50-59	12,323	16.4%	3,643	14.2%	3,823	15.8%	4,857	19.1%	
60-69	22,636	30.1%	7,403	28.9%	7,337	30.3%	7,896	31.0%	
70-79	25,016	33.2%	9,201	36.0%	8,144	33.6%	7,671	30.1%	
80+	10,442	13.9%	4,113	16.1%	3,481	14.4%	2,848	11.2%	
**Gender**									
Male	46,447	61.7%	15,956	62.4%	14,881	61.5%	15,610	61.2%	p=0.018
Female	28,833	38.3%	962	37.6%	9,332	38.5%	9,881	38.8%	
**Hospitalization in the year prior to the index surgery**									
0	49,883	66.3%	17,685	69.1%	16,256	67.1%	15,942	62.5%	p<0.001
1	17,308	23.0%	5,651	22.1%	5,480	22.6%	6,177	24.2%	
>1	8,089	10.7%	224	8.8%	2,477	10.2%	3,372	13.2%	
**Abdominal surgery in the 3 years prior to the index surgery**									
No	70,685	93.9%	24,003	93.8%	22,714	93.8%	23,968	94.0%	p=0.560
Yes	4,595	6.1%	1573	6.2%	1499	6.2%	1,523	6.0%	
**Charlson score**									
0	60,011	79.7%	20,274	79.3%	19,210	79.3%	20,527	80.5%	p<0.001
1 - 2	13,816	18.4%	4,822	18.9%	4,487	18.5%	4,507	17.7%	
3+	1,453	1.9%	480	1.9%	516	2.1%	457	1.8%	
**Year of index Hospitalization**									
2002 - 2006	26,987	35.8%	10,127	39.6%	8,269	34.2%	8,591	33.7%	p<0.001
2007 - 2010	24,491	32.5%	8,117	31.7%	7,706	31.8%	8,668	34.0%	
2011 - 2014	23,802	31.6%	7,332	28.7%	8,238	34.0%	8,232	32.3%	
**Stoma creation during the index hospitalization**									
Yes	27,306	36.3%	8,481	33.2%	9,287	38.4%	9,538	37.4%	p<0.001
No	47,974	63.7%	17,095	66.8%	14,926	61.6%	15,953	62.6%	
**Surgical approach**									
Open	58,901	78.2%	21,118	82.6%	18,079	74.7%	1,9704	77.3%	p<0.001
Laparoscopy	16,379	21.8%	4,458	17.4%	6,134	25.3%	5,787	22.7%	

### Outcomes by hospital volume

#### In-hospital mortality

Among the entire cohort, the overall rate of in-hospital mortality was 1.3%, ranging from 0.9% to 1.6% in high- and low-volume hospitals, respectively (p<0.001). (Table 2)

**Table 2:** Short-term outcomes by hospital volume

	All patients		Low volume (1 - 12)		Medium volume (13 - 31)		High volume (32+)		
	n	column %	n	column %	n	column %	n	column %	p-value
No. of Patients	75,280	100.0	25,576	100.0	24,213	100.0	25,491	100.0	
No. of hospitals/year	8280	100.0	6534	100.0	1283	100.0	463	100.0	
**Length of stay**									
Lower median (1-12)	38,684	51.4%	10,865	42.5%	12,369	51.1%	15,450	60.6%	p<0.001
Upper median (13+)	36,596	48.6%	14,711	57.5%	11,844	48.9%	10,041	39.4%	
**Modality of discharge**									
Alive	74,307	98.7%	25,166	98.4%	23,877	98.6%	25,264	99.1%	p<0.001
Dead	973	1.3%	410	1.6%	336	1.4%	227	0.9%	
**Abdominoperineal resection**									
No	63,005	83.7%	20,624	80.6%	20,479	84.6%	21,902	85.9%	p<0.001
Yes	12,275	16.3%	4,952	19.4%	3,734	15.4%	3,589	14.1%	
**30-day readmission**									
No	68,974	92.8%	23,334	92.7%	22,088	92.5%	23,552	93.2%	p<0.001
Yes	5,333	7.2%	1,832	7.3%	1,789	7.5%	1,712	6.8%	

In the multivariate analysis, all the variables considered showed an independent impact on the in-hospital mortality (Table 3). Compared with high volume hospitals, the adjusted OR of in-hospital mortality was 49% higher in low-volume hospitals. (Table 3)

**Table 3:** Multivariate logistical regression analysis reporting the adjusted odds of each outcome

Variables	In-hospital mortality			Abdominoperineal resection			Length of stay			30-day readmission		
	OR	IC 95%		OR	IC 95%		OR	IC 95%		OR	IC 95%	
**Volume (procedures/year)**												
High (32+)	1.00			1.00			1.00			1.00		
Medium (13 - 31)	1.41	1.17	1.70	1.16	1.07	1.26	1.54	1.37	1.74	1.09	1.00	1.18
Low (1 - 12)	1.49	1.25	1.78	1.49	1.38	1.61	2.29	2.05	2.55	1.10	1.02	1.19
**Age categories (years)**												
18-49	1.00			1.00			1.00			1.00		
50-59	4.93	1.78	13.62	1.06	0.95	1.17	1.05	0.97	1.13	0.90	0.79	1.03
60-69	6.53	2.42	17.67	1.14	1.04	1.26	1.23	1.15	1.33	0.95	0.84	1.08
70-79	15.31	5.70	41.08	1.46	1.32	1.60	1.70	1.58	1.83	1.00	0.88	1.13
80+	30.47	11.33	81.92	1.68	1.51	1.86	2.31	2.12	2.50	1.21	1.05	1.38
**Gender**												
Male	1.00			1.00			1.00			1.00		
Female	0.66	0.57	0.76	1.01	0.97	1.06	0.89	0.86	0.92	0.79	0.75	0.84
**Hospitalization in the year prior to the index surgery**												
0	1.00			1.00			1.00			1.00		
1	1.12	0.96	1.31	1.48	1.41	1.55	1.16	1.11	1.21	1.20	1.12	1.29
>1	1.39	1.15	1.69	2.00	1.87	2.13	1.32	1.25	1.40	1.27	1.16	1.40
**Abdominal surgery in the 3 years prior to the index surgery**												
No	1.00			1.00			1.00			1.00		
Yes	1.47	1.20	1.80	0.87	0.80	0.95	1.02	0.95	1.10	1.10	0.98	1.23
**Charlson score**												
0	1.00			1.00			1.00			1.00		
1 - 2	1.43	1.23	1.67	0.98	0.92	1.03	1.27	1.21	1.33	1.29	1.20	1.39
3+	3.60	2.80	4.63	0.85	0.73	0.98	1.62	1.43	1.84	1.82	1.54	2.16
**Year of index Hospitalization**												
2002 - 2006	1.00			1.00			1.00			1.00		
2007 - 2010	0.80	0.68	0.94	0.83	0.78	0.89	0.61	0.56	0.66	1.15	1.06	1.24
2011 - 2014	0.71	0.60	0.84	0.85	0.80	0.91	0.40	0.37	0.43	1.34	1.24	1.45
**Stoma creation during the index hospitalization**												
No	1.00			1.00			1.00			1.00		
Yes	1.04	0.91	1.19	0.72	0.69	0.76	1.53	1.47	1.58	1.62	1.53	1.72
**Surgical approach**												
Open	1.00			1.00			1.00			1.00		
Laparoscopy	0.47	0.38	0.59	0.71	0.67	0.76	0.54	0.51	0.56	1.07	1.00	1.15

#### Abdominoperineal resection

Among the entire cohort, the overall rate of APR was 16.4%, ranging from 14.1% to 19.4% in high- and low-volume hospitals, respectively (p<0.001). (Table 2)

All the explicative variables except gender were independently associated with the APR rate. The probability of undergoing an APR was 49% higher in patients operated on in low-volume hospitals, as compared to high-volume hospitals. (Table 3)

#### Length of stay

Overall, the median (Interquartile Range) LOS was 13 (10-19) days. It was ≥13 days in 39.4% and in 57.5% of cases in high- and low-volume hospitals, respectively (p=0.001). (Table 2)

With the exclusion of the abdominal surgery performed in the 3 years prior to the index surgery, the remaining variables were independently associated with the LOS. (Table 3)

#### Thirty-day readmission

Overall, the rate of 30-day readmission was 7.1%, ranging from 6.8% to 7.3% in the high- and low-volume hospitals, respectively (p=0.001). (Table 2)

Compared with high-volume hospitals, the adjusted OR of 30-day readmission was 10% higher in low-volume hospitals.

The HV, along with age, gender, hospitalization in the year prior to the index surgery, Charlson score, and study period was independently associated with high rates of 30-day readmission (Table 3).

### Variation of hospital volume and outcomes during the study period

**Figure 2:**
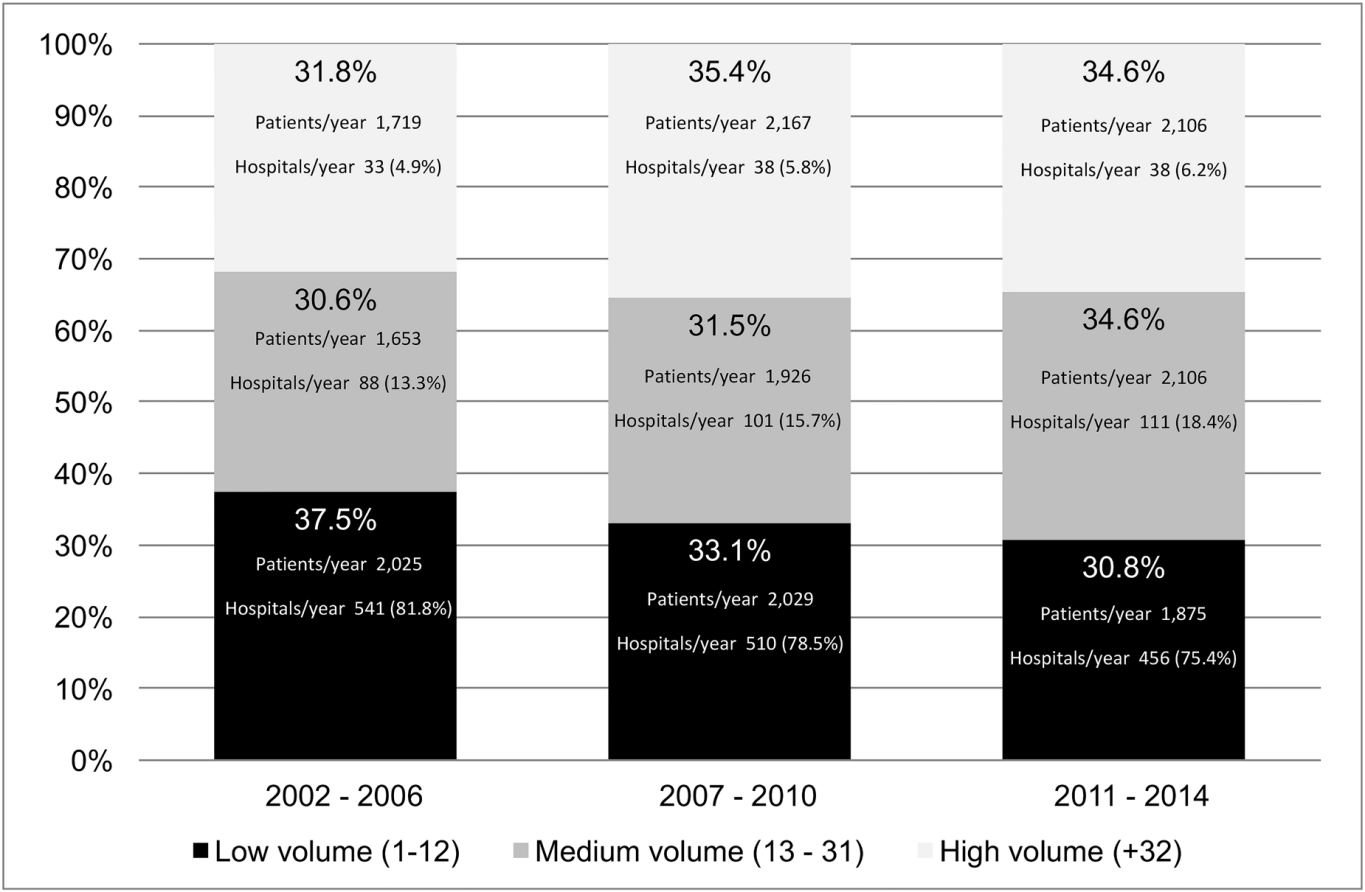
Distribution of patients according to the hospitals' annual volume of procedures, by period

Across the study period, both the proportion of patients who underwent surgery in low-volume hospitals and the proportion of hospitals that performed less than 13 procedures/year decreased, indicating an ongoing and “spontaneous” process of centralization of rectal surgery. (Figure 2) In detail, the proportion of patients treated in low-volume hospitals decreased by 6.7 percent, from 37.5% in 2002-2006 to 30.8% in 2011-2014, while the proportion of low-volume hospitals decreased by 6.4 percent, from 81.8% in 2002-2006 to 75.4% in 2011-2014.

The proportion of patients with stoma creation during index hospitalization significantly increased during the study period: 31.5% in 2002-2006, 36.1% in 2007-2010 and 41.8% in 2011-2014 (p<0.001).

**Figure 3:**
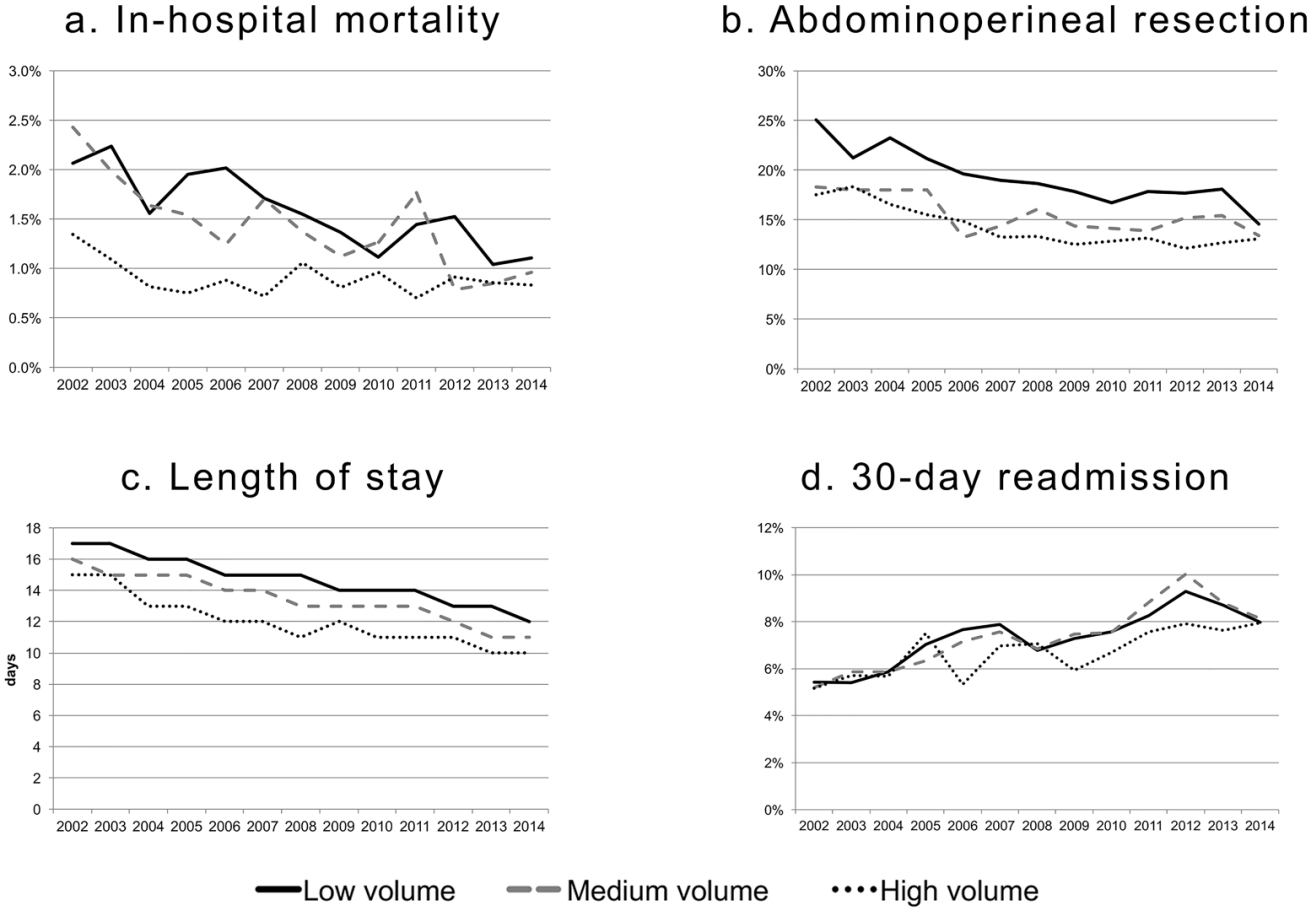
Temporal trends of the outcome measures according to the hospitals' annual volume of procedures

Among the four outcome measures considered, the rates of the in-hospital mortality, APR, and the LOS decreased overtime, while the rate of 30-day readmission increased. This trend was similar for each HV category. (Figure 3)

## DISCUSSION

The principal aim of this study was to investigate the association between HV and some of the most used short-term outcomes after major elective rectal cancer surgery. APR resection was included in the outcomes as it is widely considered to reflect the ability of the surgeon to preserve the anal sphincters and, like postoperative mortality, has been suggested to be one of top scored colorectal cancer care quality measures [10].

The main finding of the study was that the risk of a worse outcome was significantly higher among patients who underwent surgery in low-volume than in those who underwent surgery in high-volume hospitals. This association was independent from the other covariates and was found for all the outcomes.

A second finding, made possible by the longitudinal design of the study and already observed by others [11], was the shift over time of patients admitted to high-volume hospitals. As, throughout the study period, no specific laws, policy recommendations or guidelines were adopted to influence such a process, it suggests that a process of centralization is “spontaneously” underway.

Comparisons between studies on this topic are challenging and many factors should be taken in account to explain differences in findings. Differences in the inclusion/exclusion criteria, definition of perioperative mortality and HV, study design, data source, study period, specificity of different healthcare systems, and cultural and geographical aspects may largely explain discrepancies between study results. A specific consideration should be reserved to postoperative mortality, which is a rare event in rectal cancer. In their large population-based study, Bilimoria et al. [2] evaluated perioperative mortality in seven common malignancies. The rates of postoperative mortality for rectal cancer were the lowest among all other malignancies (1.9% in the highest and 3.0% in the lowest volume-hospitals, respectively). The corresponding figures for other malignancies were respectively: 5% and 5.9% (colon), 6.1% and 10.9% (esophagus), 6.2% and 11.9% (liver), 5.5% and 6.4% (lung), 4.9% and 10.5% (pancreas), and 5.7% and 8.9% (stomach). Based on these figures, the sample size powered to find statistically significant differences in postoperative mortality after rectal cancer surgery is crucial [12]. The overall rate of in-hospital mortality in our study was 1.3%, which is in the range (0.79% to 5.2%) of that reported by others [3, 13–15]; it was 0.9% and 1.6% in the highest and lowest HV tertile, respectively. With a such a small difference, only studies with a large sample size are able to capture a statistically significant difference. The only study to include more than 50,000 cases showed results similar to ours [2]. The rates of in-hospital mortality decreased during the study period for each HV tertile; nevertheless, like others [2, 16–19], we found that the HV still remained an independent risk factor for in-hospital mortality. Opposite findings were reported by studies based on smaller sample sizes [4, 13–15, 18, 20, 21].

Despite the exclusion of cancers located at the recto-sigmoid junction, we found an APR rate of 16%, which is lower than that reported by most of the other studies [3, 14–16, 21, 22]. This is not surprising, because cultural aspects likely play a relevant role for this specific outcome, and may explain this discrepancy. Other explanations rely on the study period. Studies reporting on series of patients operated in the last century show rates of APR of more than 50% [21–23]. In addition, the rate of sphincter-preserving procedures does not exactly reflect the rate of patients who are stoma-free. A proportion of patients, challenging to define, who underwent sphincter-preserving surgery either had the stoma never closed or underwent a stoma creation as a consequence of an anastomotic leak. Compared to perioperative mortality, the association between HV and the rate of APR or permanent colostomy is less debated and the majority of authors report findings similar to ours [6, 9, 11, 15–20, 23–27], while only a few disagree [14, 28, 21].

We found a median LOS of 13 days which decreased overtime. While the LOS varies between studies due to differences in healthcare systems and cultural attitude [13, 15], a decreasing LOS over time is a largely observed phenomenon as a consequence of cost-containment policies and of the improvement of available home health care and skilled facilities. Interestingly, Balentine et al. [28] reported a greater likelihood of discharge to home for colorectal cancer patients admitted in high-volume vs. low-volume hospitals. There is large agreement on the favorable impact of HV on this outcome [6, 13, 15, 17].

The association of the 30-day readmission and HV is frequently reported for colorectal cancer, while few studies specifically report on this relationship after major elective rectal cancer surgery. Our finding of 7.2% of 30-day readmission compares favorably with 10.7% reported by Doumouras e al. [29] Similar to Schneider et al. [30], we found that the risk of readmission increased across the study periods. In the multivariate analysis, being operated on in more recent time periods independently increased the odds of readmission. Some temporal factors might have had an impact on this finding. Neoadjuvant therapy has been increasingly used and is currently standard of care for mid-low locally advanced rectal cancer; however, it is also considered a potential risk factor of postoperative morbidity. In addition, the proportion of patients having a covering stoma during the study period increased from 31.5% in 2002-2006 to 41.8% in 2011-2014. As postoperative morbidity and stoma-related problems have been found to impact negatively on 30-day readmission [29, 30], it is reasonable to explain the increased rates of 30-day hospitalization with the increased use of neoadjuvant treatments and covering stoma. Furthermore, early discharge is thought to have an impact on readmissions; however, several studies found either no impact or an inverse association between LOS and the rate of 30-day readmission [29, 30].

The main strength of this study is the large sample size, the national-based source of data and the evaluation of multiple outcomes. The database does not contain any missing data which guarantees consistency of the analyses performed. Furthermore, the longitudinal design of the study produced a picture of variation over time of treatments and outcomes. Finally, unlike older studies, this analysis refers to a study period when neoadjuvant therapy, total mesorectal excision technique and the laparoscopic approach were widely diffused.

Limitations of the study are mainly related to the data source. Given its administrative nature, the data-base used does not allow for risk stratification. Clinical data such as tumor size, stage, tumor distance from the anal sphincter, or the use of adjuvant radiation or chemotherapy all of which may have an impact on short-term outcomes are not available. Likewise, the surgeon who performed the procedure is not identifiable and therefore the impact of surgeon volume on the outcomes is lacking. Although in some studies [21], the impact of surgeon volume on the outcomes has been found to be more relevant than HV, the latter appears to be an appropriate surrogate for surgeon volume in colorectal resections [31]. A further weakness is related to the lack of reliable data on postoperative complications. We excluded this outcome because it has been demonstrated that the information and coding on postoperative complications are often omitted or under-reported. [32] Moreover, we could not report on long-term outcomes (i.e., overall and disease-free survival) because a link with databases with information on the vital status of patients was not feasible.

Finally, the translation of our findings in different healthcare systems should be verified but it is possible that proposed observations are generalizable to countries with a healthcare system similar to ours.

In conclusion, we report convincing evidence that hospital volume is independently associated with short-term outcomes in the setting of the elective complex rectal cancer surgery. These findings do support the hypothesis that a process of centralization might improve outcomes. Such a process is spontaneously underway, without the aid of policy decisions or guideline recommendations.

## MATERIALS AND METHODS

### Study design and data source

This was a retrospective, longitudinal, national-based cohort study. The data were retrieved from the administrative National Italian Hospital Discharge Dataset, which was established in 1996 and is currently utilized by the Italian Ministry of Health for administrative purposes (reimbursement of hospitals based on the Diagnosis-Related Group system). A national annual report on hospital admissions is available on-line for epidemiological studies; furthermore, the Ministry supplies researchers with anonymized data from the database [33]. Healthcare in Italy is universally delivered by public funds. The hospitals included in the national health system received the accreditation by the National Ministry of Health. This guarantees a standard in the medical treatment among them. The accreditation system has been adopted in Italy since 1992.

For the aims of this study, Ministry of Health provided data on admissions that took place from January 1, 2000 to December 31, 2014.

The hospital discharge form reports on patient demographics, date of admission, surgical procedures and discharge. It codes for one primary and five secondary diagnoses and up to six performed procedures, surgical approach (open or laparoscopic), acuity of the admission (emergent, urgent or elective), and status at discharge (dead or alive). It does not report on stage of disease, distance of the tumor from the anal verge, preoperative or postoperative chemo and/or radiotherapy, and identification of the surgeon who performed the procedure. Moreover, information on overall and disease-free survival through linkage with other databases was not possible.

After approval of the study design by the Italian Ministry of Health, we had access to the data for this specific study. The analysis and interpretation of the data are the sole responsibility of the authors.

### Patient selection and definitions

Patients were identified according to the International Classification of Diseases, Ninth Revision, Clinical Modification 2007 (ICD-9-CM). The inclusion criteria were: age 18+ years, diagnosis of primary rectal cancer (ICD9-CM 154.x), major surgical procedure (ICD9-CM codes: 45.8, 45.95, 48.49, 48.5, 48.61-48.69) performed between January 2002 and November 2014. The available records regarding hospital admissions during 2000-2001 were used to exclude cases with a prevalent procedure for rectal cancer by January 1, 2002; the records regarding hospital admissions that took place in December 2014 were used to determine the 30-day readmission of patients with a hospital admission up to November 30, 2014.

The exclusion criteria were: prevalent procedure for rectal cancer before January 1, 2002, cancer of the anus (154.2-154.3) or of the recto-sigmoid junction (ICD9-CM 154.1), minor rectal cancer procedures, placement of a stoma before the index hospitalization and discharge to acute-hospitals if the record of the second hospitalization was unavailable. Patients who died during the index hospitalization were excluded from the analysis of the 30-day readmission.

### Outcome measures

The outcome measures were: in-hospital mortality, which was defined as death due to any cause during the index hospitalization; rate of APR, which was the proportion of APR on the total major rectal procedures; LOS of the index hospitalization, which was defined as the difference between date of discharge and date of admission; and 30-day readmission, which was defined as any unplanned, distinct hospitalization within 30 days after the discharge of the index hospitalization.

These outcomes were chosen because they are widely used to measure the quality of healthcare and are easily retrieved from administrative databases; moreover, unlike other information such as postoperative morbidity, recording of these outcomes is mandatory and less frequently burdened by coding errors.

### Hospital volume and additional covariates

Hospital volume was calculated as the average annual number of rectal cancer procedures performed at each hospital during the study period. We defined the thresholds of volume tertiles calculated on the whole study population and the hospitals were then categorized as low, medium and high volume for each study year accordingly.

The following additional covariates were assessed for the prediction of the outcomes of interest: age (subdivided into four classes: 18-59, 60-69, 70-79, and 80+ years), gender (male, female), indexes of surgical complexity or comorbidity (non-colorectal surgery-related hospitalizations in the year prior to the index hospitalization, admissions for abdominal non-colorectal cancer-related surgery and the Charlson Index both referring to the three years prior to the index hospitalization [34], year of the index hospitalization (subdivided into three classes: 2002-2006, 2007-2010, 2011-2014), creation of stoma during the index hospitalization, and open or laparoscopic approach. Of course, 30-day readmission was calculated only on those patients who were alive at discharge.

### Statistical analysis

The chi-square test was used to assess differences in demographics and clinical characteristics between hospitals with different levels of annual procedure volumes (low, medium and high).

Multivariate logistical regression was used to calculate the adjusted odds ratio (OR) for each of the four study outcomes (in-hospital mortality, APR, LOS, and 30-day readmission). Multilevel regression was utilized to account for the hierarchical structure of the data (first level: patient; second level: hospital). In the multilevel analysis, the LOS was categorized into two levels, i.e., under and over the median.

Statistical significance was set at p<0.05. Stata software was used to perform all analyses (Stata Corporation, Stata Statistical Software: Release 13.0. College Station, TX).

### Limitations and biases

The study is based on administrative data. Tumor size, stage, tumor distance from the anal sphincter, adjuvant radiation or chemotherapy, surgeon who performed the procedure, postoperative complications records are not present and analyzed. The database used has no link with other databases containing clinical information. The risk of biases is related to the absence of this information.

Manuel Zorzi and Nicola Gennaro participated in study conception, research design, acquisition, statistical analysis, interpretation of data and drafting of the manuscript. They were responsible for relationship with Italian Ministry of Health.

Francesco Marchegiani, Andrea Barina, Matteo Zuin, Alessandro Perin and Patrick Frambach participated in research design, acquisition, analysis, interpretation of data, drafting and critical revision of the manuscript.

Massimo Rugge, Isacco Maretto, Francesca Bergamo, Caterina Boso and Emanuele Damiano Luca Urso participated in research design, acquisition, analysis, interpretation of data and critical revision of the manuscript.

Maria Chiara Corti participated in research design, acquisition and statistical analysis of the data. She obtained the authorization to data access from Italian Ministry of Health. She participated in drafting and revision of the manuscript.

All the mentioned authors gave final approval of the version to be published.

## Abbreviations

APR: abdominoperineal resection
CI: confidence interval
HV: hospital volume
LOS: length of stay
OR: odds ratio
